# Purkinje-cell-specific DNA repair-deficient mice reveal that dietary restriction protects neurons by cell-intrinsic preservation of genomic health

**DOI:** 10.3389/fnagi.2022.1095801

**Published:** 2023-01-24

**Authors:** María Björk Birkisdóttir, Lisanne J. Van’t Sant, Renata M. C. Brandt, Sander Barnhoorn, Jan H. J. Hoeijmakers, Wilbert P. Vermeij, Dick Jaarsma

**Affiliations:** ^1^Department of Neuroscience, Erasmus MC, Rotterdam, Netherlands; ^2^Princess Máxima Center for Pediatric Oncology, Utrecht, Netherlands; ^3^Oncode Institute, Utrecht, Netherlands; ^4^Department of Molecular Genetics, Erasmus MC Cancer Institute, Erasmus University Medical Center Rotterdam, Rotterdam, Netherlands; ^5^Faculty of Medicine, CECAD, Institute for Genome Stability in Aging and Disease, University of Cologne, Cologne, Germany

**Keywords:** cerebellum, DNA repair, nucleotide excision repair, transcription stress, dietary restriction, Progeria, Purkinje neurons, DNA damage

## Abstract

Dietary restriction (DR) is a universal anti-aging intervention, which reduces age-related nervous system pathologies and neurological decline. The degree to which the neuroprotective effect of DR operates by attenuating cell intrinsic degradative processes rather than influencing non-cell autonomous factors such as glial and vascular health or systemic inflammatory status is incompletely understood. Following up on our finding that DR has a remarkably large beneficial effect on nervous system pathology in whole-body DNA repair-deficient progeroid mice, we show here that DR also exerts strong neuroprotection in mouse models in which a single neuronal cell type, i.e., cerebellar Purkinje cells, experience genotoxic stress and consequent premature aging-like dysfunction. Purkinje cell specific hypomorphic and knock-out ERCC1 mice on DR retained 40 and 25% more neurons, respectively, with equal protection against P53 activation, and alike results from whole-body ERCC1-deficient mice. Our findings show that DR strongly reduces Purkinje cell death in our Purkinje cell-specific accelerated aging mouse model, indicating that DR protects Purkinje cells from intrinsic DNA-damage-driven neurodegeneration.

## Introduction

Dietary restriction (DR, also known as caloric restriction) is a long-term dietary intervention that consists of a daily 15 to 40% reduction of food intake without malnutrition, and that is well known as a universal anti-aging intervention increasing health-and lifespan (Le Couteur and Simpson, 2018; Lee et al., 2021; Green et al., 2022). DR reduces age-related decline in motor and cognitive function, as well as nervous system pathologies associated with normal aging and age-related neurodegenerative diseases (Mattison et al., 2017; Xie et al., 2020). While the benefits of DR as an anti-aging intervention are well established, the mechanisms by which aging is counteracted are still largely unresolved (Speakman and Mitchell, 2011; Lee et al., 2021). On the one hand, aging itself is a complex multifactorial phenomenon with numerous detrimental processes occurring at cellular (accumulation of damaged macromolecules and intracellular debris), organ (e.g., impaired tissue renewal), as well as whole organism level (changes in hormonal, serological and inflammatory status; Lopez-Otin et al., 2013; Pluvinage and Wyss-Coray, 2020). On the other hand, DR affects numerous intra- and intercellular processes, which include nutrient sensing, metabolic, hormonal and immunoregulatory pathways (Speakman and Mitchell, 2011; Green et al., 2022). Thus, a major challenge in understanding the anti-aging mechanisms of DR lies in connecting the pathways modulated by DR with the variety of aging processes that operate distinctively across tissues and cell types (Speakman and Mitchell, 2011; Ma et al., 2020; Schaum et al., 2020; Vougioukalaki et al., 2022).

We have previously found that DR has a remarkably strong beneficial effect on life-and health span in progeroid *Ercc1^Δ/−^* mice, in which defective DNA repair results in chronic genome instability and numerous features of multimorbidity and accelerated aging (Vermeij et al., 2016a). These data show that DR may alleviate the detrimental effects of impaired DNA repair, and indicate that DR protects against genotoxic stress, which is one of the driving factors of aging (Lopez-Otin et al., 2013). Most notably, DR strongly reduced the dramatic, progressive neurodegeneration (Vermeij et al., 2016a). Since *Ercc1^Δ/−^* mice develop systemic multimorbidity, with severely compromised functions of vital organs, blood vessels and immune system (Vermeij et al., 2016a,b; Vougioukalaki et al., 2022), a key question remained whether DR is neuroprotective indirectly *via* local and/or systemic components (e.g., by reducing inflammation), or by directly lowering cell-intrinsic DNA-damage-driven neurotoxicity. This question is particularly relevant for the central nervous system in view of increasing evidence favoring a central role of systemic factors in nervous system aging and neurodegeneration (Pluvinage and Wyss-Coray, 2020). Therefore, we examined the effect of DR in a mouse model in which DNA repair deficiency drives accelerated aging only in one single neuronal cell type, i.e., cerebellar Purkinje cells. These neurons are severely affected, but largely protected by DR, in whole-body *Ercc1^Δ/−^* mice (Birkisdottir et al., 2021), and degenerate in human aging (Andersen et al., 2003) as well as in various genome instability disorders (Shiloh, 2020; Kwak et al., 2021).

## Materials and Methods

### Ethic statements

Animal experiments were performed according to institutional guidelines as overseen by the Animal Welfare Board of the Erasmus MC, following Dutch and EU legislation. Prior to the start of the experiments, a project license for the animal experiments performed for this study was obtained from the Dutch national authority and filed under no. AVD101002015273 (DEC no. 139-12-13, 139-12-18, 15-273-120, 15-273-159).

### Mice

*Ercc1*^∆/−^, *Pcp2-Ercc1^Δ/f^*, and *Pcp2-Ercc1^f/−^* were generated as previously described (de Waard et al., 2010; de Graaf et al., 2013; Vermeij et al., 2016a; Van't Sant et al., 2021). To obtain *Ercc1*^∆/−^ mice, we crossed *Ercc1*^∆/+^ with *Ercc1*^+/−^ mice (both in pure FVB or C57BL6J backgrounds respectively) to yield *Ercc1*^∆/−^ offspring with a genetically uniform C57BL6J/FVB F1 hybrid background (Weeda et al., 1997; Vermeij et al., 2016a). In these mice one allele is fully inactivated whereas the other shows reduced activity, caused by a seven amino acids truncation. Because the *Ercc1*^∆/−^ mice were smaller, food was administered within the cages, and water bottles with long nozzles were used from around 2 weeks of age.

For the generation of Purkinje specific *Pcp2-Cre^+^/Ercc1*^∆/*f*^ mice (named *Pcp2-Ercc1^Δ/f^* in this study), *Pcp2-Cre^+^/Ercc1*^∆*/+*^ mice in C57Bl6/J background were generated by crossing female *Pcp2-Cre^+^* mice with male *Ercc1*^∆/*+*^ mice. Subsequently, female *Pcp2-Cre^+^/Ercc1*^∆*/+*^ were crossed with male *Ercc1^f/f^* mice in FVB background to yield *Pcp2-Cre^+^/Ercc1*^∆*/f*^ in a uniform C57BL6J/FVB F1 hybrid background. *Pcp2-Cre^+^/Ercc1^+/f^* and *Pcp2-Cre^−^/Ercc1*^∆*/f*^ transgenic littermates that do not develop a degenerative phenotype served as controls (Van't Sant et al., 2021). Additional controls for this study were wild-type animals from the same C57BL6J/FVB F1 hybrid genetic background. Typical unfavorable characteristics, such as blindness in an FVB background or deafness in a C57BL6J background, do not occur in this hybrid background.

For initial characterization of cerebellar motor deficits and Purkinje cell degeneration two different Pcp2-Cre lines were used: Mpin Pcp2-Cre (Barski et al., 2000) and Jdhu Pcp2-Cre (Zhang et al., 2004). We used the Pcp2-Cre Mpin line for the DR experiments.The Pcp2-Cre Mpin line also was used to generate *Pcp2-Ercc1^−/f^* (*Pcp2-Cre^+^/Ercc1*^−/*f*^) mice. These were generated similarly as the above described *Pcp2-Ercc1^Δ/f^* mice, but by introducing an *Ercc1* allele that is fully inactivated (−) rather than truncated (Δ) from the initial breedings (de Graaf et al., 2013).

### Housing conditions and dietary regimens

Animals of DR experiments were randomly divided over sex-matched dietary groups and housed in individual ventilated cages under specific pathogen-free conditions. Each cohort consisted of four groups (experimental/control genotype and AL/DR diet) of at least four animals, thereof two males and two females. Animals of other behavioral experiments were group housed. Environment was controlled with a temperature of 20–22°C and 12 h light:12 h dark cycles. Food and water were offered *ad libitum* (AL) unless otherwise stated. Mice were weighed, visually inspected weekly, and scored blindly for gross morphological and motor abnormalities weekly. Animals were bred and maintained on AIN93G synthetic pellets (Research Diet Services B.V., Wijk bij Duurstede, Netherlands; gross energy content 4.9 kcal/g dry mass, digestible energy 3.97 kcal/g). The amount of DR was determined in a prior pilot study where food intake of the AL-fed mice was continuously monitored. Mice on average ate 3.0 g food per day, resulting in 2.1 g/day for 30% DR. Water was freely available. Food was given to the animals just before the start of the dark (active) period (ZT12). DR was initiated at 7 weeks of age with 10% food reduction, and food was gradually reduced to 30% DR from 9 weeks of age onward as previously published (Vermeij et al., 2016a). Our DR regimen in a room with altered day-night rhythm was specifically chosen not to disturb the biological clock, as this may influence the anti-aging effect of DR (Nelson and Halberg, 1986; Acosta-Rodriguez et al., 2022).

### Behavioral assays

To examine cerebellar motor function, we used an accelerating rotarod, balance beam and Erasmus automated ladder test, largely following previously described protocols (Vinueza Veloz et al., 2015; Vermeij et al., 2016a; White et al., 2021) and further detailed in the Supplementary methods.

### Histological procedures

Mice were anaesthetized with pentobarbital and perfused transcardially with 4% paraformaldehyde. Cerebella were carefully dissected out, post-fixed for 1 h in 4% paraformaldehyde and left in sucrose overnight. Subsequently the brain was embedded in 12% gelatin, rapidly frozen, and sectioned at 40 μm using a freezing microtome. Sections were processed free floating using single-, double-, and triple-labelling immunofluorescence. Primary antibodies (supplier; catalogue number, RRID number; dilution) used in this study were as follows: rabbit anti-Calbindin (Swant; C9638, AB_2314070; 1:20,000); mouse anti-Calbindin (Sigma Aldrich; C8666, AB_2313712; 1:20,000); goat anti-FOXP2 (Santa Cruz Biotechnology; sc21069, AB_2107124; 1:1000); rabbit anti-GFAP (DAKO; Z0334, AB_10013382; 1:8000); rat anti-LGALS3 (Cedarlane; CL8942AP, AB_10060357; 1:1000) rabbit anti-P53 (Leica; NCL-p53-CMP5, AB_2744683; 1:1000) and mouse anti-GM130 (BD Bioscience; 610,822, AB_398141; 1:200). Alexa488-, Cy3-, and Cy5-conjugated antibodies raised in donkey (Jackson ImmunoResearch) diluted at 1:200 were used as secondary antibodies for visualization. Immunofluorescence sections were analyzed and imaged using Zeiss LSM700, Leica TCS SP8, and Leica Stellaris 5 confocal microscopes, and a Zeiss Axio Imager D2 widefield microsope.

### Stereological analysis of Purkinje cell numbers

Stereological quantification of Purkinje neurons was done as previously described (Birkisdottir et al., 2021) using the Optical Fractionator tool of a StereoInvestigator software package (MBF Bioscience), integrated in a Zeiss LSM700 confocal microscope setup (see Supplementary methods). Our estimates of the number of Purkinje cells in control mice (from 2.04*10^5^ to 2.48*10^5^; Supplementary Figure S3A) were consistent with stereological quantifications of other studies (Woodruff-Pak et al., 2010; Birkisdottir et al., 2021).

### Quantitative histological analyses

To determine the number of P53+ Purkinje cells, we double stained every 8^th^ serial coronal section (8–11 section/animal) for Calbindin and P53 (see histological procedures). Purkinje neurons with strongly P53-immunostained nucleus (Supplementary Figure S1G) were systematically counted throughout the whole series using a Zeiss Axio Imager D2 widefield fluorescence microscope equipped with a 40x EC Plan-NeoFluar lens. The total number of P53+ Purkinje cells/ animal was estimated by multiplying the number of counted cells by 8. To determine the proportion of P53+ cells, we used the Purkinje cell numbers from the stereological analyses.

Golgi apparatus morphological abnormalities were analyzed in the cohort of 40-week-old AL vs. DR treated *Pcp2-Ercc1^Δ/f^* mice. Coronal sections of cerebellum were double immunostained with Calbindin and the cis-Golgi protein GM130 (see histological procedure). Stacks of the Purkinje cell layer, 0.5–1 mm in length and 15 μm thick, were randomly sampled at high resolution in lobules of the vermis and paravermis using a Leica Stellaris 5 confocal microscope with a HC PL APO CS2 63x oil objective. Stacks were examined in ImageJ (Version 1.52) by a trained observer unaware of genotype and diet (DJ). Only Purkinje cells whose cell body was fully embedded in the stack were counted. In control Purkinje cells, GM130 staining appears as a ribbon-like structure that forms an extensive network throughout the cell body. In most Purkinje cells of *Pcp2-Ercc1^Δ/f^* mice GM130 staining showed a normal or near normal appearance. Purkinje cells with abnormal Golgi apparatus either showed fragmentation of the ribbon into smaller elements resulting in punctate GM130 staining, or compaction and redistribution of the Golgi ribbon to a small part of the cell body. Percentages of Purkinje cells with abnormal Golgi were determined by analyzing 100–200 Purkinje cells per AL animal (*n* = 4), and 500–700 Purkinje cells per DR animal.

To assess differences in astrocytosis, images of GFAP stained cerebellar tissue were acquired using a wide field microscope Zeiss Axio Imager D2 widefield microscopes, and mean intensities of the molecular layer of the cerebellum were measured using ImageJ.

### Statistical analyses

Statistical analyses were performed using GraphPad Prism Software (San Diego, CA, United States). For the analysis of difference between Purkinje neurons, P53 and Golgi abnormalities, values of AL and DR animals of each cohort was compared using two-tailed unpaired *t*-test. One way ANOVA with Tukey’s multiple comparison was used for GFAP analysis, Purkinje cell amount and relative rescue analysis. GFAP intensity values of AL and DR animals of each cohort were compared using two-tailed unpaired *t*-test. Two-way repeated measure ANOVA was used for rotarod data and mixed effect model (REML) for bodyweight data, both with post-hoc Tukey’s Multiple comparison test. Graphs illustrate individual values, means and standard error (SE). Statistical analyses and results are summarized in Supplementary Table S1.

## Results

**Figure 1:**
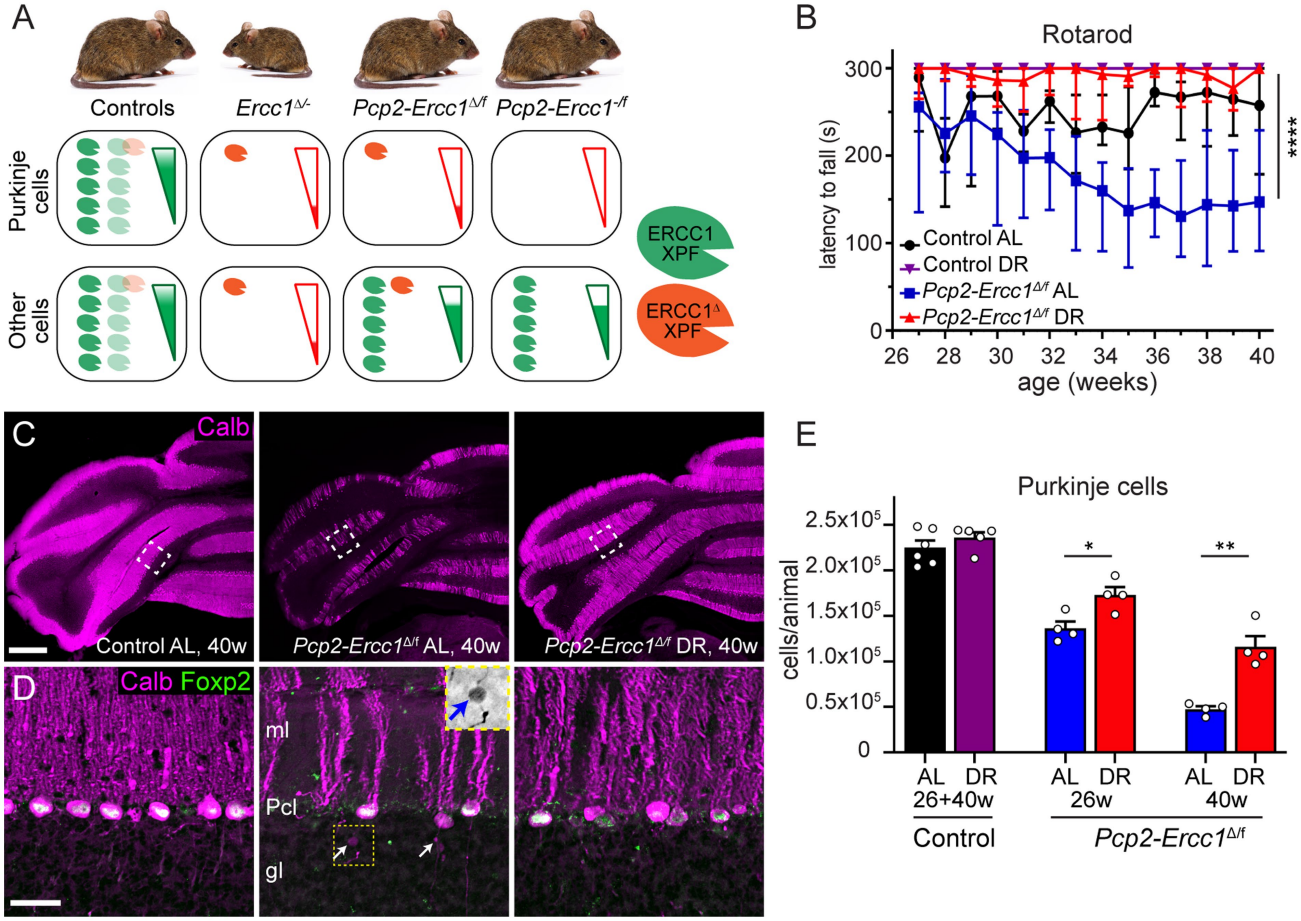
Dietary restriction reduces Purkinje cell loss in Purkinje cell specific ERCC1-deficient mice. **(A)** Schematic representation of Ercc1-XPF nuclease expression in Purkinje cells versus other cells in the Ercc1 mouse models of this study. The truncated *Ercc1^Δ^* allele results in strongly reduced levels of ERCC1^Δ^-XPF nuclease dimer (one red symbol) as compared to wild-type *Ercc1^+^* allele (5 green symbols). *Ercc1*^Δ/−^ mice have low mutant ERCC1 production in their whole body, whereas the hypomorphic mutation in the *Pcp2-Ercc1^Δ/f^* mice is restricted to Purkinje cells. In comparison, *Pcp2-Ercc1^−/f^* mice have no ERCC1 production in Purkinje cells, whereas the remainder of the body in both conditional mutants is repair-proficient due to the flox-allele that encodes wt protein. The genotype and ERCC1-XPF levels in littermates used as control animals for each cohort differ as indicated with light colored heterodimers (see methods). **(B)** Accelerating rotarod assay showing preserved performance in DR versus AL *Pcp2*-*Ercc1^Δ/f^* mice over time. Values represent medians with interquartile range (*n* = 4/group); ****p < 0.0001 (Dunn’s post-test; *Pcp2*-*Ercc1^Δ/f^* AL versus DR). **(C,D)** Low **(C)** and high magnification **(D)** confocal images of calbindin (magenta) and FOXP2 (green) immunostaining to outline Purkinje cells and their nucleus. Note, reduced loss of Purkinje cells in DR-*vs* AL-treated *Pcp2*-*Ercc1^Δ/f^* mice. Insert in middle panel in d shows an increased-contrast inverted-signal image of the axonal spheroid indicated by the yellow arrow in the same panel. gl, granular layer; ml, molecular layer; Pcl, Purkinje cell layer. **(E)** Bar graph with means ± SE of stereological quantification of Purkinje cells showing loss of Purkinje cells in 26-and 40-week-old *Pcp2*-*Ercc1^Δ/f^* mice and reduced loss in DR vs. AL animals. (*n* ≥ 4/group); *p < 0.05, **p < 0.01 (unpaired *t*-tests for each cohort). Scale bars: 500 μm **(C)**, 25 μm **(D)**.

We generated a Cre-lox-based Purkinje cell-specific ERCC1-deficient (*Pcp2-Ercc1^Δ/f^*) mouse model that carried a floxed *Ercc1* allele (*Ercc1*^f^), a C-terminally truncated “delta” *Ercc1* allele (*Ercc1^Δ^*; Vermeij et al., 2016b), and a Pcp2-Cre transgene (Mpin line) that drives Cre recombinase expression in postnatal Purkinje cells (Van't Sant et al., 2021). After recombination by Cre, the floxed *Ercc1* allele becomes a null allele, resulting in Purkinje cells that have one null and one truncated *Ercc1* allele, and that reproduce the severe (but incomplete) reduction in ERCC1/XPF nuclease function of whole body *Ercc1^Δ/−^* mice (Figure 1A), affecting nucleotide excision repair, inter-strand crosslink repair, and single-strand annealing of double-strand breaks (Vermeij et al., 2016b; Mulderrig and Garaycoechea, 2020; Apelt et al., 2021). *Pcp2-Ercc1^Δ/f^* mice had, unlike *Ercc1^Δ/−^* mice, the same weight as control littermates and did not show evidence of neurodegenerative changes outside the cerebellum, consistent with the remainder of the body and nervous system being repair proficient and healthy (Figure 1A; Supplementary Figures S1A,F). *Pcp2-Ercc1^Δ/f^* mice at 12 weeks showed control performance in cerebellar motor tests, but developed progressive deficits thereafter (Supplementary Figures S1B–D). The time of onset and severity of cerebellar motor abnormalities as well as the severity of Purkinje cell degeneration were the same as in *Pcp2-Ercc1^Δ/f^* mice generated with another Pcp2-Cre transgene (Jhdu line), indicating that the Purkinje cell degenerative phenotype is not influenced by the Cre line (Supplementary Figure S1). Since the BAC construct of the Jdhu Pcp2-Cre line may introduce extra copies of genes implicated in neuronal function, splicing and DNA repair (Pnpla6, Stxbp2, Pcp2, and Xab2; Zhang et al., 2004) we used the Pcp2-Cre Mpin line for the DR experiments.

**Figure 2:**
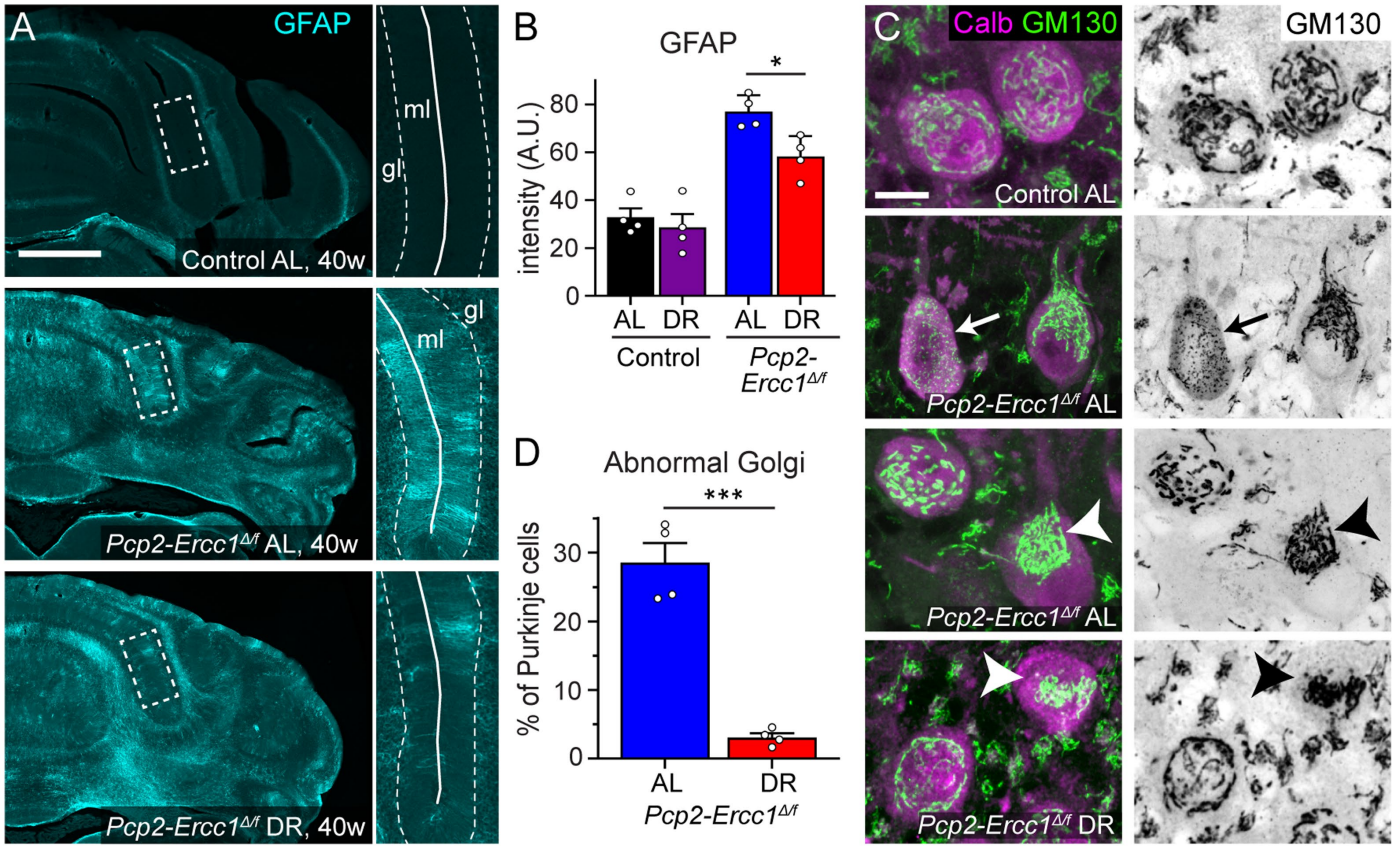
Dietary restriction reduces GFAP-immunostaining and Golgi apparatus abnormalities in *Pcp2-Ercc1^Δ/f^* mice. **(A,B)** Representative images and bar graph (means ± SE) of GFAP immunofluorescence illustrating increased GFAP immunostaining in *Pcp2*-*Ercc1^Δ/f^* cerebellum compared to control, and reduced GFAP-staining in the molecular layer (ml) of DR compared to AL *Pcp2*-*Ercc1^Δ/f^* cerebellum. (*n* = 4/group); *p < 0.05 (unpaired *t*-test). **(C,D)** Confocal images and bar graph (means ± SE) showing reduced amount of Golgi apparatus morphological abnormalities in 40 week old DR compared to AL *Pcp2*-*Ercc1^Δ/f^* Purkinje cells, examined using immunostaining of the cis-Golgi scaffold protein GM130. In control Purkinje cells the GM130 staining outlines a network of linear profiles throughout the cell body. In ERCC1-deficient mice 2 main types of changes occur in a subset of Purkinje cells: (1) fragmentation of linear profiles into smaller elements (arrow) and (2) more frequently, redistribution of unfragmented or fragmented Golgi stacks to part of the cell body (arrow heads). (*n* = 4/group); ***p < 0.001 (unpaired *t*-test). Scale bars: 500 μm **(A)**, and 10 μm **(C)**.

We applied the same 30% DR regimen as used before in full-body *Ercc1^Δ/−^* mice starting with 10% restriction at the age of 7 weeks, 20% at 8 weeks, and 30% thereafter (Vermeij et al., 2016a). DR-treated *Pcp2-Ercc1^Δ/f^* animals showed the same weight loss as DR control mice (Supplementary Figure 2A), and displayed preserved performance in an accelerating rotarod test up to the age of 40 weeks (Figure 1B). In contrast, *ad libitum* (AL)-fed *Pcp2-Ercc1^Δ/f^* mice showed strongly declining rotarod performance till 35 weeks, after which the descent appears to stabilize (Figure 1B), concomitant with severe Purkinje cell degeneration throughout the cerebellar cortex as revealed by reduced immunohistological staining for calbindin that in the cerebellum is specifically expressed in Purkinje cells (Figures 1C,D; Supplementary Figures S2B). DR-treated *Pcp2-Ercc1^Δ/f^* mice at 40 weeks, instead, showed relatively preserved calbindin staining (Figures 1C,D; Supplementary Figure S2B), indicative of reduced Purkinje cell loss. We quantified Purkinje cells using a stereological approach (Andersen et al., 2003; Birkisdottir et al., 2021), and confirmed that Purkinje cell loss was significantly (*p* < 0.01) prevented by DR with 1.16 ± 0.12 *10^5^ and 0.47 ± 0.03 *10^5^ residual Purkinje cells in 40-week-old DR-and AL-*Pcp2-Ercc1^Δ/f^* mice, respectively. Additionally, Purkinje cells of DR-treated *Pcp2-Ercc1^Δ/f^* mice showed a lower frequency of morphological abnormalities, such as axonal spheroids (Figure 1D) and displayed less astrocytosis, indicated by GFAP staining, in the molecular layer consistent with reduced Purkinje cell degeneration (Figures 2A,B; de Waard et al., 2010; de Graaf et al., 2013). Next, we analyzed Golgi apparatus morphology of Purkinje cells, following up on our finding that in *Ercc1^Δ/−^* mice DR strongly reduced the frequency of spinal motor neurons with atypical Golgi, putatively representing a proxy of compromised neuronal health (de Waard et al., 2010; Vermeij et al., 2016a). Indeed, AL-fed *Pcp2-Ercc1^Δ/f^* mice showed a spectrum of abnormal Golgi in about 25% of residual Purkinje cells, while this was strongly diminished in DR-treated *Pcp2-Ercc1^Δ/f^* mice (Figures 2C,D). In sum, DR significantly reduces motor dysfunction and Purkinje cell pathology and loss in *Pcp2-Ercc1^Δ/f^* mice at 40 weeks.

**Figure 3:**
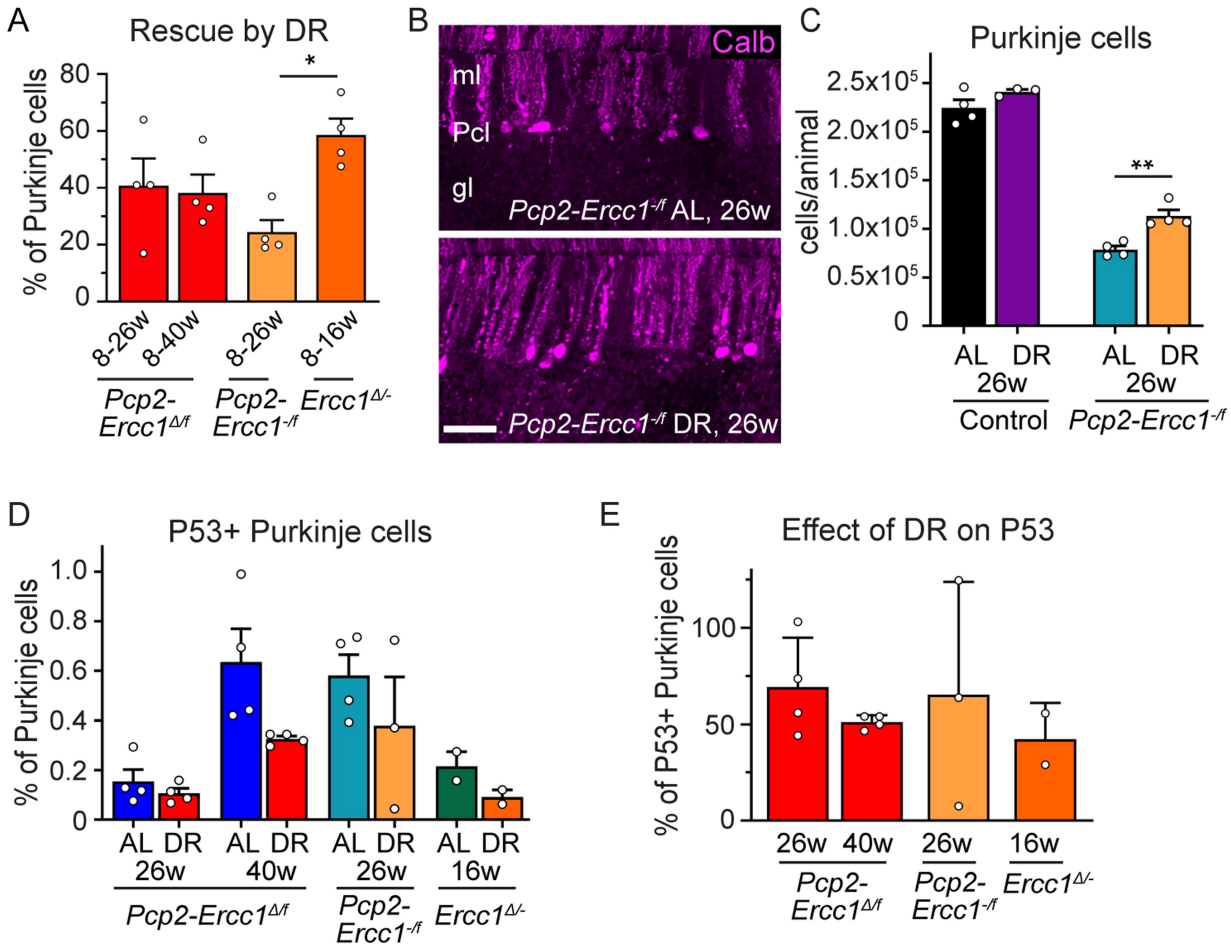
Differential protective effects of DR in Purkinje cell-specific and ubiquitous ERCC1-deficient mice. **(A)** Relative rescue of Purkinje cells by DR in Purkinje cell-specific (*Pcp2*-*Ercc1^Δ/f^* and *Pcp2*-*Ercc1^−/f^*) and global (*Ercc1^Δ/−^*) mice, calculated by dividing the difference in Purkinje cell loss between DR and AL animals by the loss showed in AL mice. (*n* = 4 per group); *p < 0.05 (One-way ANOVA; Tukey’s post-tests). **(B,C)** Representative confocal images **(B)** and bar graph **(C)** illustrating Purkinje cell degeneration in AL and DR *Pcp2*-*Ercc1^−/f^* mice. (*n* = 4 per group) **p < 0.01 (One-way ANOVA; Tukey’s post-test). **(D)** Bar graph of the proportion of Purkinje cells with P53-immunoreactive nuclei in ERCC1-deficient mouse mutants (*n* ≥ 2 per group). Impact of DR across all groups: *p* = 0.024 [2-way ANOVA *F*(1,16) = 6.019]; DR-AL pair comparisons: *p* = 0.0784 in AL vs. DR Pcp2-*Ercc1^Δ/f^* at 40 weeks; *p* > 0.4 in other AL-DR pairs (Sidak’s multiple comparison post-test). **(E)** Proportion of Purkinje cells with nuclear P53 accumulation expressed as % of mean AL values. Scale bar: 50 μm.

To robustly quantify the effect of DR on ERCC1-deficiency in Purkinje cells in time, we focused on stereological analysis of Purkinje cell numbers. At 8 weeks of age at the start of DR treatment, the number of Purkinje cells in *Pcp2-Ercc1^Δ/f^* mice appeared unaltered compared to controls of 8–40 weeks (2.28 ± 0.11 *10^5^ vs. 2.27 ± 0.06 *10^5^, respectively; Supplementary Figure S3). Accordingly, *Pcp2-Ercc1^Δ/f^* mice at 8 weeks showed no evidence of astrocytosis (Supplementary Figure S3D). At the age of 26 weeks, DR and AL mice showed 1.73 ± 0.09 * 10^5^ and 1.36 ± 0.08 *10^5^ remaining Purkinje cells, respectively (Figure 1E) revealing a significant protective effect of DR (*p* < 0.05). Using the mean value at 8 weeks (2.28 *10^5^) as starting number of Purkinje cells in *Pcp2-Ercc1^Δ/f^* mice (Supplementary Figure S3B), we estimated the number of Purkinje cells that died in AL and DR animals at 26 and 40 weeks (= 2.28 *10^5^- # of surviving Purkinje cells). Subsequently, these values were used to calculate a relative rescue of Purkinje cells by DR with the formula: (average loss in AL animals – loss in DR animal)/average loss in AL animals*100. This analysis indicated that DR attenuated Purkinje cell loss by about 40% at both ages (Figure 3A). This percentage appears somewhat smaller than the 59% estimated relative rescue of Purkinje cell loss by DR for full-body *Ercc1^Δ/−^* mice, examined at the age of 16 weeks, a few weeks before the progeroid mutants become moribund (Figure 3A; Birkisdottir et al., 2021), albeit the difference did not reach statistical significance (*p* = 0.289 and 0.188 for 26 and 40 weeks respectively). It should be noted, that systemic *Ercc1^Δ/−^* mice do not only differ from *Pcp2-Ercc1^Δ/f^* in regard of ubiquitous versus Purkinje-cell-specific ERCC1-deficiency, but also show earlier onset of ERCC1 cell deficiency (early development versus post-natal week 2), and already exhibit Purkinje cell loss at the onset of DR treatment at 8 weeks (Supplementary Figure S3B).

Hypomorphic *Pcp2*-*Ercc1^Δ/f^* and full-body *Ercc1^Δ/−^* mice still have some residual repair activity, which may contribute to the protective effect of DR. Therefore, we tested DR in *Pcp2-Ercc1*^−/f^ mice that are fully ERCC1-deficient in Purkinje cells (Figure 1A). *Pcp2-Ercc1*^−/f^ mice show unaltered numbers of Purkinje cells at 8 weeks (Supplementary Figure S3B), like *Pcp2*-*Ercc1^Δ/f^* mice and in accord with previous proteomic and neuropathological data (de Graaf et al., 2013). However, they display a larger (*p* < 0.0001) Purkinje cell loss at 26 weeks with 0.78 ± 0.07 *10^5^ (vs. 1.36 ± 0.04 *10^5^ for Pcp2-*Ercc1^Δ/f^* mice) residual Purkinje cells, and only sparse surviving Purkinje cells at 40 weeks (0.23 ± 0.03 *10^5^; Supplementary Figures S3B,C), indicating a dose–response relationship between degree of repair deficiency and neuronal cell loss. As in *Pcp2*-*Ercc1^Δ/f^* mice, DR significantly (*p* < 0.01) reduced Purkinje cell loss in *Pcp2-Ercc1*^−/f^ mice at 26 weeks of age (Figures 3B,C), with a relative rescue of about 25% compared to AL animals (Figure 3A). These data indicate that DR also is protective in Purkinje cells with complete loss of ERCC1, that experience more severe genotoxic stress than *Ercc1^Δ/−^* Purkinje cells (Figure 1A).

To get insight into the role of DNA damage we examined P53 as guardian of the genome. Previously, we found a decrease in P53-expressing cells in the neocortex and spinal cord of DR *Ercc1^Δ/−^* mice, supporting that DR reduces DNA damage load (Vermeij et al., 2016a). Here, we also observed an overall reduction [*p* = 0.024; 2-way ANOVA F(1,16) = 6.019] by DR on P53 positive Purkinje neurons, albeit the difference between each individual AL-DR pair not being statistically significant (Figure 3D). Across all models and cohorts, DR equally reduced the proportion of Purkinje cells with nuclear P53 accumulation by about 50% (Figure 3E).

## Discussion

In this study we show that DR strongly reduces degeneration of ERCC1-deficient Purkinje cells, in mice that are unaffected in other cell types and tissues. The data imply that DR strongly reduces cell-intrinsic DNA-damage driven Purkinje cell degeneration. The data also indicate that reduced Purkinje cell degeneration by DR in whole-body ERCC1-deficient mice primarily results from cell intrinsic mechanisms rather than by restoring function of surrounding glia cells and vital organs or *via* systemic components. Our data support previous work indicating that DR may both reduce the levels of DNA damage and increase the ability of cells to cope with DNA damage (Heydari et al., 2007), and support the hypothesis that the anti-aging effect of DR in the nervous system, at least in part, is mediated by reducing genotoxic stress in neurons (Vermeij et al., 2016a; Xie et al., 2020).

Previously, we have shown that ERCC1-deficient mice develop multiple age-dependent neurological deficits and progressive neuropathology characterized by a diversity of degenerative changes throughout the nervous affecting both neurons and glia (de Waard et al., 2010; Borgesius et al., 2011; Vegh et al., 2012; Raj et al., 2014; Vermeij et al., 2016a). The nervous system pathology is consistent with a model where stochastic accumulation of DNA damage causes transcription stress, i.e., a variety of yet incompletely defined transcription problems and down-stream effects that can affect cell function in multiple ways, for instance by blocked or deregulated expression of essential genes or problems resulting from persistent transcription stalling (Vermeij et al., 2016b; Niedernhofer et al., 2018; Lans et al., 2019; Mulderrig and Garaycoechea, 2020; Apelt et al., 2021; Jia et al., 2021; Van't Sant et al., 2021). Here, we focused on stereological analysis of residual Purkinje cell numbers as the principal readout, representing an unequivocal quantitative readout that reflects the final outcome of multiple neurodegenerative pathways. In addition, we show that DR has impact on two additional readouts in Purkinje cells: it reduces the frequency of Purkinje cells expressing the genotoxic stress transcription factor P53, and Purkinje cells with abnormal Golgi apparatus morphologies. The varied perturbations in Golgi morphology in more than 20% of ERCC1-deficient Purkinje cells and their strongly reduced frequency in DR animals, are consistent with findings in spinal motor neurons in *Ercc1^Δ/−^* mice (de Waard et al., 2010; Vermeij et al., 2016a), and may reflect a diversity of degenerative and cell stress pathways including DNA damage signaling (Makhoul et al., 2019).

The identity of DNA lesions that accumulate in ERCC1-deficient nervous system and cause neuronal damage are yet poorly understood (Niedernhofer et al., 2018; Lans et al., 2019; Mulderrig et al., 2021). In our previous DR study with *Ercc1^Δ/−^* mice, analyses of liver transcriptomes showed a preferential reduction in expression of long genes in *Ercc1^Δ/−^* liver, which was attenuated by DR (Vermeij et al., 2016a). This is consistent with the notion that long genes have an increased likelihood of accumulating transcription stalling lesions (Lans et al., 2019; Noe Gonzalez et al., 2021), and display reduced damage in DR animals. Hence, analysis of gene-length-dependent transcriptional changes reflect a promising indirect readout for measuring transcription stress and the effect of DR in *Ercc1^Δ/−^* nervous system. Importantly, a bias of reduced expression of long genes also has been found in aged human nervous system (Vermeij et al., 2016a; Niedernhofer et al., 2018; Soheili-Nezhad et al., 2021), indicating that DNA-damage-induced transcription stress is an important factor contributing to nervous system aging and targeted by DR (Vermeij et al., 2016a). Future studies employing high resolution spatial transcriptomic approaches (Asp et al., 2020; Lewis et al., 2021) may uncover the relationships between transcriptional abnormalities and neuronal degeneration in aging and progeroid models.

What changes triggered by DR render Purkinje cells less vulnerable towards DNA repair deficiencies? DR has been shown to promote autophagy and to reduce ribosomal protein S6 phosphorylation in Purkinje cells, indicative of suppression of mTOR signalling (Alirezaei et al., 2010), one of the main pathways linked to DR benefits (Liu and Sabatini, 2020; Green et al., 2022). However, we recently showed that mTOR inhibition *via* rapamycin or genetic intervention fails to improve lifespan, neurological functioning, and Purkinje cell survival of *Ercc1^Δ/−^* mice (Birkisdottir et al., 2021), hence, pointing to alternative mechanisms. While a spectrum of other potential anti-aging compounds that have been proposed to reproduce aspects of the beneficial effects of DR by influencing nutrient sensing, inflammation, mitochondrial function, or glucose homeostasis, did not have an effect on survival and neurological function in *Ercc1^Δ/−^* mice, we found that two NAD^+^ precursors (nicotinamide riboside and nicotinic acid) induced small benefits in survival and neurological function (Alyodawi et al., 2019; Birkisdottir et al., 2022). Since DR may increase NAD^+^ levels and modulate a variety of NAD^+^-dependent mechanisms in energy and redox metabolism, as well as in DNA damage sensing, protein deacetylation or other enzymatic pathways requiring NAD^+^ (Covarrubias et al., 2020; Green et al., 2022), NAD^+^-dependent pathways may be part of the mechanisms mediating the beneficial effect of DR in ERCC1-deficient Purkinje cells. However, further studies are needed to dissect the mechanisms that underlie the strong beneficial effect of DR in ERCC1-deficient mice. Our Purkinje cell specific ERCC1-deficient mice provides a complementary model to global *Ercc1^Δ/−^* mice in these studies.

## Data availability statement

The original contributions presented in the study are included in the article/Supplementary material, further inquiries can be directed to the corresponding author/s.

## Ethics statement

The animal study was reviewed and approved by Animal Welfare Board of the Erasmus MC, ErasmusMC Rotterdam. Written informed consent was obtained from the owners for the participation of their animals in this study.

## Author contributions

WV and DJ conceptualized and designed the experiments. MB, LS, WV, and DJ wrote the manuscript and performed statistical analysis of data. JH provided critical advice, and contributed to writing and editing of the manuscript. RB, SB, and WV planned and performed mouse experiments. MB, LS, and DJ performed histopathological assessments. All authors contributed to the article and approved the submitted version.

## Funding

We acknowledge financial support of the National Institute of Health (NIH)/National Institute of Aging (NIA) (AG17242), European Research Council Advanced Grants Dam2Age (to JH), ONCODE supported by the Dutch Cancer Society, ADPS Longevity Research Award (to WV), Memorabel (ZonMW 733050810), BBoL (NWO-ENW 737.016.015), Deutsche Forschungsgemeinschaft (DFG, German Research Foundation—Project-ID 73111208—SFB 829), Regiodeal Foodvalley (162135), and the European Joint Programme Rare Diseases (TC-NER RD20-113).

## Conflict of interest

The authors declare that the research was conducted in the absence of any commercial or financial relationships that could be construed as a potential conflict of interest.

## Publisher’s note

All claims expressed in this article are solely those of the authors and do not necessarily represent those of their affiliated organizations, or those of the publisher, the editors and the reviewers. Any product that may be evaluated in this article, or claim that may be made by its manufacturer, is not guaranteed or endorsed by the publisher.
