# Supplementary material for: Purkinje-cell-specific DNA repair-deficient mice reveal that dietary restriction protects neurons by cell-intrinsic preservation of genomic health
Source: Front Aging Neurosci. 2023 Jan 24;14:1095801. doi: 10.3389/fnagi.2022.1095801 (PMC9902592; doi:10.3389/fnagi.2022.1095801)
Supplement: Supplementary file 1 [file Table_1.pdf]

Table 1. Statistical analyses. Included are all test performed, including post-hoc test that reach significance. Bold p values are significant

	% of variation	Sum of squares	DF	Test's statistic*	P Value
**Fig 1B. Rotarod**					
Two-way RM ANOVA: Time x Cohort on Performance					
Weeks x Cohort	8.414	76593	39	2505	**<0,0001**
Row factor (Weeks)	2.281	20766	13	2037	0.09260
Column factor (Cohort)	58.47	532216	3	13,44	**0.00040**
Subject	17.4	158391	12	16,84	**<0,0001**
Residual		122306	156		
Kruskal- Wallis Test					
Performance all groups				50.75	**<0,0001**
Dunn's multiple comparisons test (Post Hoc)					
Pcp2-Ercc1$^{\Delta/f}$ AL vs.Pcp2-Ercc1$^{\Delta/f}$ DR				4.54	**<0,0001**
Pcp2-Ercc1$^{\Delta/f}$ AL vs. Con DR				6.669	**<0,0001**
Con AL vs. Con DR				4.633	**<0,0001**
**Fig 1E. Purkinje cells**					
Unpaired t test (two-tailed)					
Con AL vs DR			9	1088	0.30510
Pcp2-Ercc1Δ/f 26wks AL vs DR			6	3186	**0.01890**
Pcp2-Ercc1Δ/f 40wks AL vs DR			6	5597	**0.00140**
**Fig 2B. GFAP**					
Unpaired t test (two-tailed)					
Con AL vs DR			6	0,6249	0.55510
Pcp2-Ercc1Δ/f 40wks AL vs DR			6	3419	**0.01420**
**Fig 2D. Golgi abnormalities**					
Unpaired t test (two-tailed)					
Pcp2-Ercc1Δ/f 40wks AL vs DR			6	8699	**0.00010**
**Fig 3A. Rescue of Purkinje cells by DR**					
Ordinary One way ANOVA					
Treatment (between columns)		2645	3	4824	**0,0199**
Residual (within columns)		2193	12		
Total		4837	15		
Tukey's multiple comparison (Post Hoc)					
Ercc1Δ/- vs.Pcp2-Ercc1-/f			12	5.359	**0.01190**
**Fig 3C. Purkinje cells in Pcp2-Ercc1-/f**					
Unpaired t test (two-tailed)					
Con AL vs DR			5	1660	0.15790
Pcp2-Ercc1-/f 26w AL vs DR			6	4759	**0.00310**
**Fig 3D. P53 in Purkinje cells**					
Two-way RM ANOVA: Diet x Cohort on %P53					
Diet x Cohort	4.383	0.07309	3	0,8109	0.50350
Row Factor (diet)	10.84	0.1808	1	6019	**0.02400**
Column Factor (cohort)	46.76	0.7797	3	8651	**0.00080**
Residual		0.5708	19		
Sidak's multiple comparison test (Post Hoc)					
Pcp2-Ercc1Δ/f 26wks AL vs DR			19	0.386	0.9923
Pcp2-Ercc1Δ/f 40wks AL vs DR			19	2.534	0.0784
Pcp2-Ercc1-/f 26w AL vs DR			19	1.518	0.4667
Ercc1Δ/-16w AL vs DR			19	0.7186	0.9275
**Fig 3E. Relative rescue by DR on p53**					
Ordinary One way ANOVA					

Treatment (between columns)		1341	3	0,4362	0,7325
Residual (within columns)		9222	9		
Total		10563	12		

	% of variation	Sum of squares	DF	Test's statistic*	P Value
**Fig S1A. Body weight males**					
Ordinary One way ANOVA					
Treatment (between columns)		8.885	3	0,5281	0,6648
Residual (within columns)		314	56		
Total		322.9	59		
**Fig S1A. Body weight females**					
Ordinary One way ANOVA					
Treatment (between columns)		21.79	3	6950	**0,0005**
Residual (within columns)		58.52	56		
Total		80.31	59		
Tukey's multiple comparison (Post Hoc)					
Mpin-control vs. Jdhu-control			56	5.942	**0.00050**
Mpin-exp vs.Jdhu-control			56	5.032	**0.00420**
**Fig S1B. Balance Beam**					
Mixed-effects model (REML)					
Weeks x Cohort			6	45,09	**<0,0001**
Row factor (Weeks)			3	83,83	**<0,0001**
Column factor (Cohort)			39	15,30	**<0,0001**
Subject	1.79				
Residual	9.276				
Tukey's multiple comparison (Post Hoc)					
wk 13 Mpin-control vs. Jdhu-exp			13.75	6.375	**0.00250**
wk 17 Mpin-exp vs. Jdhu-control			8.858	4.857	**0.03190**
wk 18 Mpin-control vs. Jdhu-exp			9.045	4.425	**0.04920**
wk 19 Mpin-control vs.Mpin-exp			8.888	7.424	**0.00250**
wk 19 Mpin-control vs. Jdhu-exp			9.15	6.085	**0.00850**
wk 19 Mpin-exp vs. Jdhu-control			16.83	4.436	**0.02810**
wk 19 Jdhu-control vs. Jdhu-exp			12.57	5.288	**0.01220**
wk 20 Mpin-control vs.Mpin-exp			8.254	6.857	**0.00510**
wk 20 Mpin-control vs. Jdhu-exp			9.139	7.276	**0.00270**
wk 20Mpin-exp vs. Jdhu-control			11.26	5.455	**0.01170**
wk 20 Jdhu-control vs. Jdhu-exp			10.82	6.352	**0.00450**
wk 21 Mpin-control vs. Mpin-exp			8.536	11.44	**0.00010**
wk 21 Mpin-control vs. Jdhu-exp			9.397	12.71	**<0,0001**
wk 21 Mpin-exp vs. Jdhu-control			8.649	11.6	**0.00010**
wk 21 Jdhu-control vs. Jdhu-exp			9.479	12.83	**<0,0001**
wk 22 Mpin-control vs. Mpin-exp			8.242	9.432	**0.00060**
wk 22 Mpin-control vs. Jdhu-exp			9.324	12.17	**<0,0001**
wk 22 Mpin-exp vs. Jdhu-control			9.356	8.558	**0.00080**
wk 22 Jdhu-control vs. Jdhu-exp			10.8	11.09	**<0,0001**
wk 23 Mpin-control vs. Mpin-exp			8.342	10.37	**0.00030**
wk 23 Mpin-control vs. Jdhu-exp			10.55	22.64	**<0,0001**
wk 23 Mpin-exp vs. Jdhu-control			8.6	10.09	**0.00030**
wk 23 Jdhu-control vs. Jdhu-exp			11.7	21.59	**<0,0001**
wk 24 Mpin-control vs. Jdhu-exp			9.385	20.69	**<0,0001**
wk 24 Mpin-exp vs. Jdhu-control			14.98	46.72	**<0,0001**

	% of variation	Sum of squares	DF	Test's statistic*	P Value
wk 24 Jdhu-control vs. Jdhu-exp			10.3	19.8	**<0,0001**
wk 25 Mpin-control vs. Mpin-exp			9.778	44.92	**<0,0001**
wk 25 Mpin-control vs. Jdhu-exp			10.26	36.05	**<0,0001**
wk 25 Mpin-exp vs. Jdhu-control			13.69	21.08	**<0,0001**
wk 25 Jdhu-control vs. Jdhu-exp			15.85	19.64	**<0,0001**

	% of variation	Sum of squares	DF	Test's statistic*	P Value
**Fig S1C. Rotarod**					
Two-way RM ANOVA: Weeks x Cohort					
Weeks x Cohort	6.849	105694	39	2621	**0,0001**
Row Factor (Weeks)	2.882	44465	13	3308	**0,0022**
Column Factor (cohort)	28.53	440320	3	10,08	**0,0001**
Subject	32.09	495154	34	14,08	**0,0001**
Residual		457049	442		
Tukey's multiple comparison (Post Hoc)					
wk 17 Mpin-control vs. Jdhu-exp			15.54	4.816	**0.01760**
wk 17 Jdhu-control vs. Jdhu-exp			17.56	5.539	**0.00530**
wk 18 Mpin-control vs. Jdhu-exp			17	4.214	**0.03820**
wk 19 Jdhu-control vs. Jdhu-exp			16.7	5.809	**0.00380**
wk 20 Mpin-control vs. Jdhu-exp			16.84	6.826	**0.00080**
wk 20 Jdhu-control vs. Jdhu-exp			17.65	5.972	**0.00270**
wk 21 Mpin-control vs. Jdhu-exp			15.16	9.493	**<0,0001**
wk 21 Jdhu-control vs. Jdhu-exp			17.29	6.56	**0.00120**
wk 22 Mpin-control vs. Mpin-exp			15.88	5.827	**0.00410**
wk 22 Mpin-control vs. Jdhu-exp			17	5.475	**0.00610**
wk 22 Mpin-exp vs. Jdhu-control			16.99	5.36	**0.00720**
wk 22 Jdhu-control vs. Jdhu-exp			17.92	5.02	**0.01120**
wk 23 Mpin-control vs. Mpin-exp			13.69	5.13	**0.01330**
wk 23 Mpin-control vs. Jdhu-exp			16.87	7.674	**0.00020**
wk 23 Mpin-exp vs. Jdhu-control			16.25	4.195	**0.04040**
wk 23 Jdhu-control vs. Jdhu-exp			17.7	6.163	**0.00200**
wk 24 Mpin-control vs. Mpin-exp			15.69	5.182	**0.01040**
wk 24 Mpin-control vs. Jdhu-exp			16.99	7.259	**0.00040**
wk 24 Mpin-exp vs. Jdhu-control			16.73	4.772	**0.01740**
wk 24 Jdhu-control vs. Jdhu-exp			18	6.757	**0.00080**
wk 25 Mpin-control vs. Mpin-exp			14.93	5.552	**0.00660**
wk 25 Mpin-control vs. Jdhu-exp			16.95	7.34	**0.00040**
wk 25 Mpin-exp vs. Jdhu-control			15.78	4.283	**0.03650**
wk 25 Jdhu-control vs. Jdhu-exp			17.96	5.804	**0.00340**
**Fig S1D. Ladder - short steps**					
Two-way RM ANOVA: Session x Cohort					
Session x Cohort	1.56	576.2	12	1991	**0.02940**
Row Factor (Session)	1.37	505.9	4	5245	**0.00060**
Column Factor (Cohort)	38.82	14340	3	8898	**0.00020**
Subject	49.44	18265	34	22,27	**<0,0001**
Residual		3280	136		
Tukey's multiple comparison: Main column effect (Post Hoc)					
Mpin-control vs. Jdhu-exp			34	4.765	**0.00970**
Mpin-exp vs. Jdhu-control			34	5.534	**0.00220**
Jdhu-control vs. Jdhu-exp			34	5.194	**0.00430**
Two-way RM ANOVA: Session x Cohort					
Session x Cohort	1.017	579.6	12	0,7838	0.66630
Row Factor (Session)	4.565	2602	4	10,55	**<0,0001**

Column Factor (Cohort)	21.69	12364	3	4236	**0.01200**
Subject	58.04	33077	34	15,79	**<0,0001**
Residual		8381	136		
	% of variation	Sum of squares	DF	Test's statistic*	P Value
Tukey's multiple comparison: Main column effect (Post Hoc)					
Mpin-exp vs. Jdhu-control			34	3.975	**0.03880**
Jdhu-control vs. Jdhu-exp			34	4.155	**0.02870**
**Fig S1D. Ladder - low rung steps**					
Two-way RM ANOVA: Session x Cohort					
Session x Cohort	0.9052	50.96	12	0.4922	0.49220
Row Factor (Session)	6.83	384.6	4	<0,0001	**<0,0001**
Column Factor (Cohort)	38.42	2163	3	<0,0001	**<0,0001**
Subject	43.04	2423	34	<0,0001	**<0,0001**
Residual		603.1	136		
Tukey's multiple comparison: Main column effect (Post Hoc)					
Mpin-control vs. Mpin-exp			34	6.113	**0.00070**
Mpin-control vs. Jdhu-exp			34	6.299	**0.00050**
Mpin-exp vs. Jdhu-control			34	4.51	**0.01550**
Jdhu-control vs. Jdhu-exp			34	4.661	**0.01180**
**Fig S1F GFAP cerebellum**					
Ordinary One way ANOVA					
Treatment (between columns)		2092	2	25,62	**0,0001**
Residual (within columns)		530.7	13		
Total		2622	15		
Tukey's multiple comparison (Post Hoc)					
Cont vs. Mpin			8.031	13	**0.00020**
Cont vs. Jdhu			9.449	13	**<0,0001**
**Fig S1F GFAP MeO**					
Ordinary One way ANOVA					
Treatment (between columns)		3.069	2	0,2189	0,8063
Residual (within columns)		91.13	13		
Total		94.2	15		
**Fig S1F. GFAP Ctx**					
Ordinary One way ANOVA					
Treatment (between columns)		15.31	2	3323	0,0831
Residual (within columns)		20.74	9		
Total		36.05	11		
**Fig S1H. P53 Cerebellum**					
Mpin-exp vs. Jdhu-exp			4	0,7372	0.5019
**Fig S2A. Body weight males Pcp2-Ercc1Δ/f and control**					
Ordinary One way ANOVA					
Treatment (between columns)		12604	3	130,2	**<0,0001**
Residual (within columns)		4389	136		
Total		16993	139		
Tukey's multiple comparison: Main column effect (Post Hoc)					
Con AL m vs. Con DR m			136	19.89	**<0,0001**
Con AL m vs. Pcp2-Ercc1Δ/f DR m			136	19.58	**<0,0001**
Pcp2-Ercc1Δ/f AL m vs. Pcp2-Ercc1Δ/f DR m			136	19.64	**<0,0001**
**Fig S2A. Body weight females Pcp2-Ercc1Δ/f and control**					
Ordinary One way ANOVA					
Treatment (between columns)		4196	3	53,46	**<0,0001**
Residual (within columns)		3558	136		

	% of variation	Sum of squares	DF	Test's statistic*	P Value
Total		7754	139		
Tukey's multiple comparison: Main column effect (Post Hoc)					
Con AL f vs. Con DR f			136	14.1	**<0,0001**
Con AL f vs. Pcp2-Ercc1Δ/f AL f			136	4.661	**0.00680**
Con AL f vs.Pcp2-Ercc1Δ/f DR f			136	14.98	**<0,0001**
Con DR f vs. Pcp2-Ercc1Δ/f AL f			136	9.439	**<0,0001**
Pcp2-Ercc1Δ/f AL f vs. Pcp2-Ercc1Δ/f DR f			136	10.32	**<0,0001**
**Fig S3A. Control animals Purkinje cells**					
Ordinary One way ANOVA					
Treatment (between columns)		2222703660	7	0,9757	0,4934
Residual (within columns)		3579627161	11		
Total		5802330820	18		
**Fig S3B. Purkinje cell numbers in AL Pcp2-Ercc1d/f, Pcp2-Ercc1-/f and Ercc1d/- mice**					
Ordinary One way ANOVA					
Treatment (between columns)		1.63466E+11	8	120,6	**0,0001**
Residual (within columns)		3896689310	23		
Total		1.67362E+11	31		
Tukey's multiple comparison (Post Hoc)					
Pcp2-Ercc1Δ/f8wks vs. Pcp2-Ercc1Δ/f26wks			23	13.01	**<0,0001**
Pcp2-Ercc1Δ/f8wks vs. Pcp2-Ercc1Δ/f40wks			23	25.63	**<0,0001**
Pcp2-Ercc1Δ/f8wks vs. Pcp2-Ercc1-/f26wks			23	21.17	**<0,0001**
Pcp2-Ercc1Δ/f8wks vs. Pcp2-Ercc1-/f40wks			23	24.31	**<0,0001**
Pcp2-Ercc1Δ/f8wks vs. Ercc1d/-8wks			23	5.867	**0.00960**
Pcp2-Ercc1Δ/f8wks vs. Ercc1d/-16wks			23	15.8	**<0,0001**
Pcp2-Ercc1Δ/f26wks vs. Pcp2-Ercc1Δ/f40wks			23	13.64	**<0,0001**
Pcp2-Ercc1Δ/f26wks vs. Pcp2-Ercc1-/f8wks			23	15.75	**<0,0001**
Pcp2-Ercc1Δ/f26wks vs. Pcp2-Ercc1-/f26wks			23	8.823	**<0,0001**
Pcp2-Ercc1Δ/f26wks vs. Pcp2-Ercc1-/f40wks			23	14.15	**<0,0001**
Pcp2-Ercc1Δ/f26wks vs. Ercc1d/-4wks			23	10.13	**<0,0001**
Pcp2-Ercc1Δ/f26wks vs. Ercc1d/-8wks			23	5.286	**0.02460**
Pcp2-Ercc1Δ/f40wks vs. Pcp2-Ercc1-/f8wks			23	30.12	**<0,0001**
Pcp2-Ercc1Δ/f40wks vs. Ercc1d/-4wks			23	23.76	**<0,0001**
Pcp2-Ercc1Δ/f40wks vs. Ercc1d/-8wks			23	16.42	**<0,0001**
Pcp2-Ercc1Δ/f40wks vs. Ercc1d/-16wks			23	10.62	**<0,0001**
Pcp2-Ercc1-/f8wks vs. Pcp2-Ercc1-/f26wks			23	25.05	**<0,0001**
Pcp2-Ercc1-/f8wks vs. Pcp2-Ercc1-/f40wks			23	27.28	**<0,0001**
Pcp2-Ercc1-/f8wks vs. Ercc1d/-4wks			23	5.078	**0.03400**
Pcp2-Ercc1-/f8wks vs. Ercc1d/-8wks			23	7.158	**0.00110**
Pcp2-Ercc1-/f8wks vs. Ercc1d/-16wks			23	18.93	**<0,0001**
Pcp2-Ercc1-/f26wks vs. Pcp2-Ercc1-/f40wks			23	6.951	**0.00160**
Pcp2-Ercc1-/f26wks vs. Ercc1d/-4wks			23	18.95	**<0,0001**
Pcp2-Ercc1-/f26wks vs. Ercc1d/-8wks			23	12.49	**<0,0001**
Pcp2-Ercc1-/f26wks vs. Ercc1d/-16wks			23	5.803	**0.01070**
Pcp2-Ercc1-/f40wks vs. Ercc1d/-4wks			23	22.42	**<0,0001**

* Tests statistics: One-Way ANOVA/ Two- Way ANOVA/ Mixed effect model (REML) = **F**; Kruskal Wallis Test = **Kruskal Wallis Statistics**; Dunn's multiple comparison test = Z; Unpaired T-test = **T** ; Tukey's multiple comparison test = **q**; Log-rank (Mantel Cox) test = **Chi Square**
