# Supplementary material for: Purkinje-cell-specific DNA repair-deficient mice reveal that dietary restriction protects neurons by cell-intrinsic preservation of genomic health
Source: Front Aging Neurosci. 2023 Jan 24;14:1095801. doi: 10.3389/fnagi.2022.1095801 (PMC9902592; doi:10.3389/fnagi.2022.1095801)
Supplement: Supplementary file 2 [file Data_Sheet_1.pdf]

Supplementary methods

Behavioural assays

To examine cerebellar motor function, we used an accelerating rotarod, balance beam and Erasmus automated ladder tests, largely following previously described protocols (Vermeij et al., 2016a; White et al., 2021). For initial characterization of *Pcp2-Ercc1*$^{\Delta/f}$ lines the accelerating rotarod and balance beam tests were performed once a week to examine the onset and progression of cerebellar motor deficits in time, while the Erasmus ladder test was performed at 26 weeks of age. In the DR vs AL cohorts, we primarily used the rotarod test.

In the Rotarod test (Ugo Basile, Varese, Italy, 7650) animals had to walk on a cylinder with a diameter of 3 cm that gradually accelerated from 2 rotations per minute (rpm) to a maximum speed of 40 rpm during 300 seconds. Performance was assessed by measuring the time spent on the rotarod until the mice were unable to follow the speed of the rod and fell off, with 300 s as the maximum time. Training consisted of four trails (1 h interval between trials) for two consecutive days. Weekly testing consisted of two trials with an interval of 1 h.

The balance beam consisted of 1-meter-long and 6 or 12 mm diameter horizontal beam, supported by a metal pole on one end and home cage on the other end. In the training sessions animals learned how to cross the beam from the platform to the cage. Test runs were recorded on video, for assessment of crossing time and the number of slips per run.

The Erasmus Ladder (Noldus, Wageningen, The Netherlands) consists of a horizontal ladder counting 37 alternating low and high rungs on each side in between two shelter boxes. In training sessions, the mice learn to cross the ladder from one shelter box to the other after a light stimulus in order to avoid an air flow. A test trial consists of 42 crossings of the ladder, and stepping patterns are determined on the basis of rung touches. Readouts in this study are step size (short versus long steps), and the frequency of 'lower' steps, i.e. steps that terminate on a lower rung (White et al., 2021).

Stereological analysis of Purkinje cell numbers

Stereological quantification of Purkinje neurons was done as previously described (Birkisdottir et al., 2021) using the Optical Fractionator tool of a StereoInvestigator software package (MBF Bioscience), integrated in a Zeiss LSM700 confocal microscope setup (see supplementary methods). Shortly, cerebellar tissue was cut serially into 40 µm coronal sections, and every 8th section was double-immunostained for Calbindin and FOXP2 to outline the cell body and nucleus of Purkinje cells, respectively (see histological procedures). Confocal stacks incorporating the entire mounted section thickness (20-35 µm) were systematically sampled using the 40× oil lens, and the systematic random sampling (SRS) tool from StereoInvestigator (sampling grid sizes of 820 × 520 µm or 740 × 470 µm). Typically, we collected around 275 stacks per animal. In each stack we counted Purkinje cells on the basis of their FOXP2+ nucleus in counting boxes of 150*150*20µm or 150*150*30 µm (depending on the thickness of the mounted section) applying the counting rules of Optical Fractionator. To obtain an estimate of the total number of Purkinje cells/animal we divided the counted number of cells by the relative fraction of tissue counted using the following formula

$$= \sum Q^{-} * \frac{t}{h} * \frac{1}{asf} * \frac{1}{ssf}$$

where Q = the counted cells; t = the section mounted thickness; h= Counting frame height; asf = area sampling fraction; and ssf = section sampling fraction.

To calculate the loss of Purkinje in the different cohorts of AL and DR *Ercc1*-mutant mouse lines we subtracted the number of residual Purkinje cell from the number of Purkinje cells at 8 weeks (2.28 *10^5 for *Pcp2-Ercc1*$^{-/\Delta}$ and *Pcp2-Ercc1*$^{-/f}$ mice; 1.78*10^5 for *Ercc1*$^{\Delta/-}$ mice; Supplementary Fig. S3b), reflecting the number of Purkinje cells at the start of DR treatment. These numbers were used to determine the relative rescue by DR using the following formula:

$$Relative\ rescue\ of\ Purkinje\ neurons = \frac{\text{Average AL cell loss} - \text{DR cell loss in each animal}}{\text{Average AL cell loss}}$$

Supplementary Figure S1

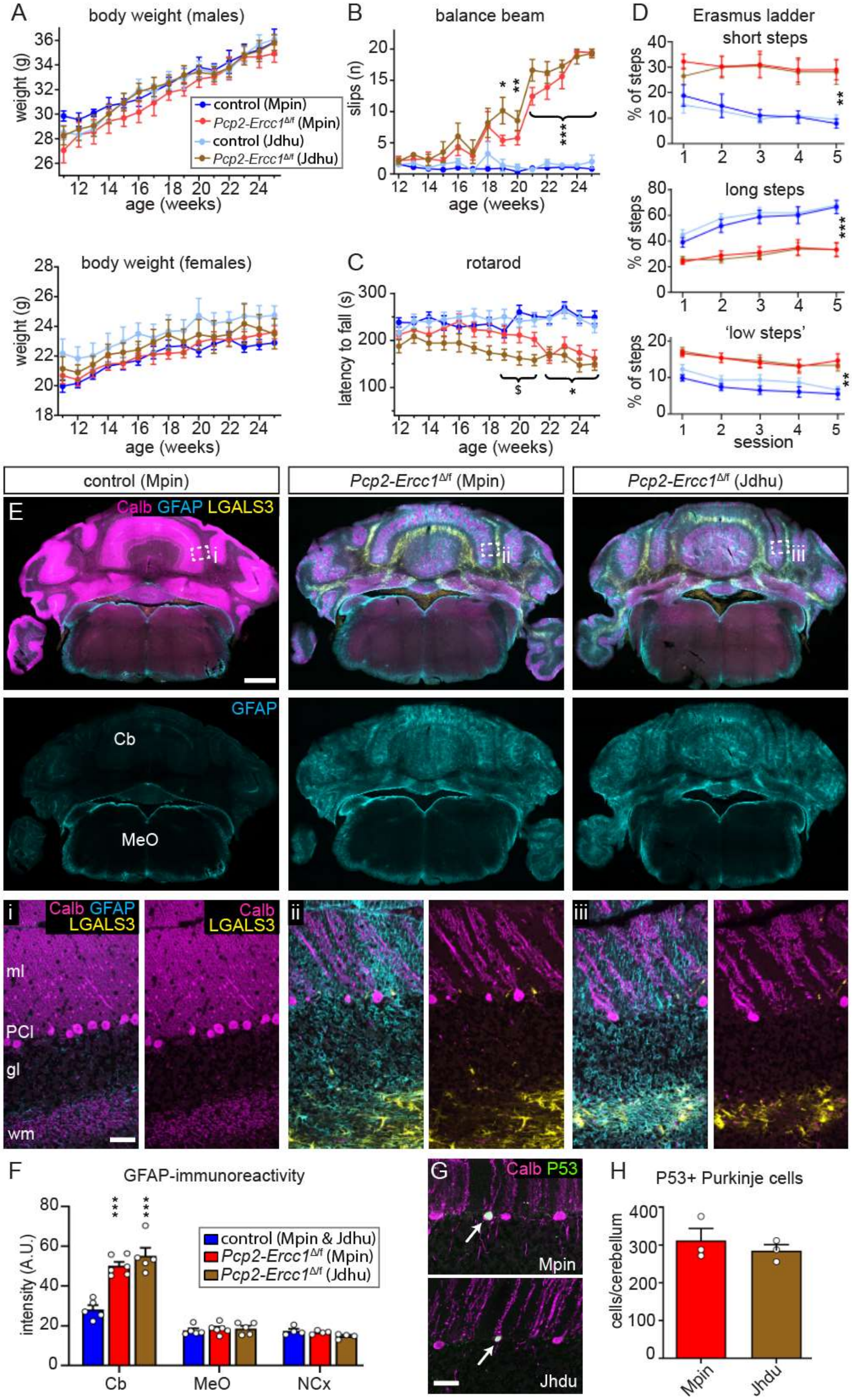

Supplementary Fig. S1 Similar neurological and neuropathological phenotypes of *Pcp2-Ercc1$^{\Delta/f}$* mice generated with Jdhu and Mpin Pcp2-Cre lines

A. Average body weight (means ± SE) of males and females over time (n ≥ 3 /group).

B-D. Mpin (3 males + 6 females; controls: 4 males + 5 females) and Jdhu (6 males + 4 females; controls: 6 males + 4 females) *Pcp2-Ercc1$^{\Delta/f}$* mice develop similar changes in cerebellar motor tests performance, showing significantly more slips compared to respective controls in balance beam test (b) from 19 weeks of age (* P<0.05,** P<0.01,*** P<0.001; Tukey's multiple comparisons post-test), and reduced performance in accelerating rotarod tests (c) from 19 weeks (Jdhu line, $ P<0.05), or 22 weeks (* P<0.05 for both Jhdu and Mpin lines). Jdhu and Mpin *Pcp2-Ercc1$^{\Delta/f}$* mice at 26 weeks produced the same increase in 'short' and 'low' step frequencies on the Erasmus ladder, consistent with severely deficient Purkinje cells (see (Vinueza Veloz et al., 2015) for interpretation of changes in step types). (** P<0.01,*** P<0.001; Tukey's multiple comparisons post-test of 2-way ANOVA for both Jdhu and Mpin line).

E. Triple staining for Calb, GFAP and LGALS3. Purkinje cell degeneration associated with appearance of LGALS3+ phagocytosing microglia cells and astrocytes is similar in Jdhu and Mpin Pcp2-Cre lines.

F. Bar graph of GFAP immunoreactivity illustrating increased GFAP immunostaining in both Mpin and Jdhu *Pcp2-Ercc1$^{\Delta/f}$* cerebellum compared to control, and no change in GFAP-staining in the medulla oblongata (MeO) and neocortex (NCx). (n≥5/group) *** P<0.001 (Tukey's multiple comparisons post-test).

G, H. Confocal images and bar graph (means ± SE) illustrating Purkinje cells with P53-immunoreactive nuclei in Mpin and Jhdu *Pcp2-Ercc1$^{\Delta/f}$* mice at 26 weeks. (n=3/group)

Scale bars: 1 mm (E), 50 µm (E, G).

Supplementary Figure S2

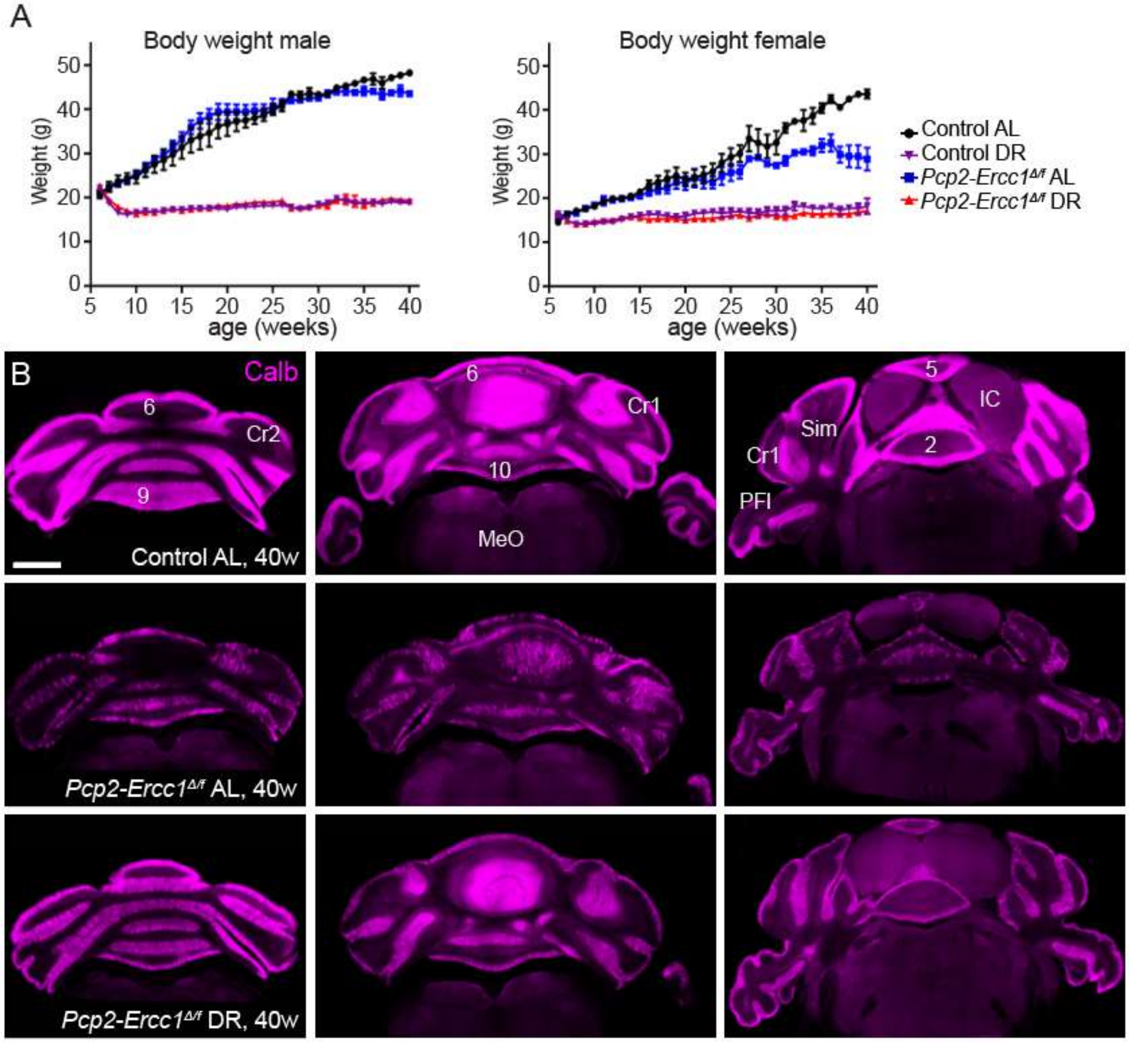

Supplementary Fig. S2. Dietary restriction reduces Purkinje cell degeneration in *Pcp2-Ercc1$^{\Delta/f}$* mice

A. Bodyweight (means ± SE) over time showing reduced weight of DR compared to AL in both *Pcp2-Ercc1$^{\Delta/f}$* and control mice. (n≥4/group)

B. Overview images of cerebellar cortex at 3 anterior-posterior levels illustrating strongly reduced calbindin-immunostaining throughout the cerebellar cortex of 40 week old AL *Pcp2-Ercc1$^{\Delta/f}$* mice and relatively preserved staining in DR *Pcp2-Ercc1$^{\Delta/f}$* mice.

Cr1, Crus 1; Cr2, Crus2; Sim, lobules simplex; PFl, paraflocculus; MeO, medulla oblongata.

Scale bars: 1 mm (B).

Supplementary Figure S3

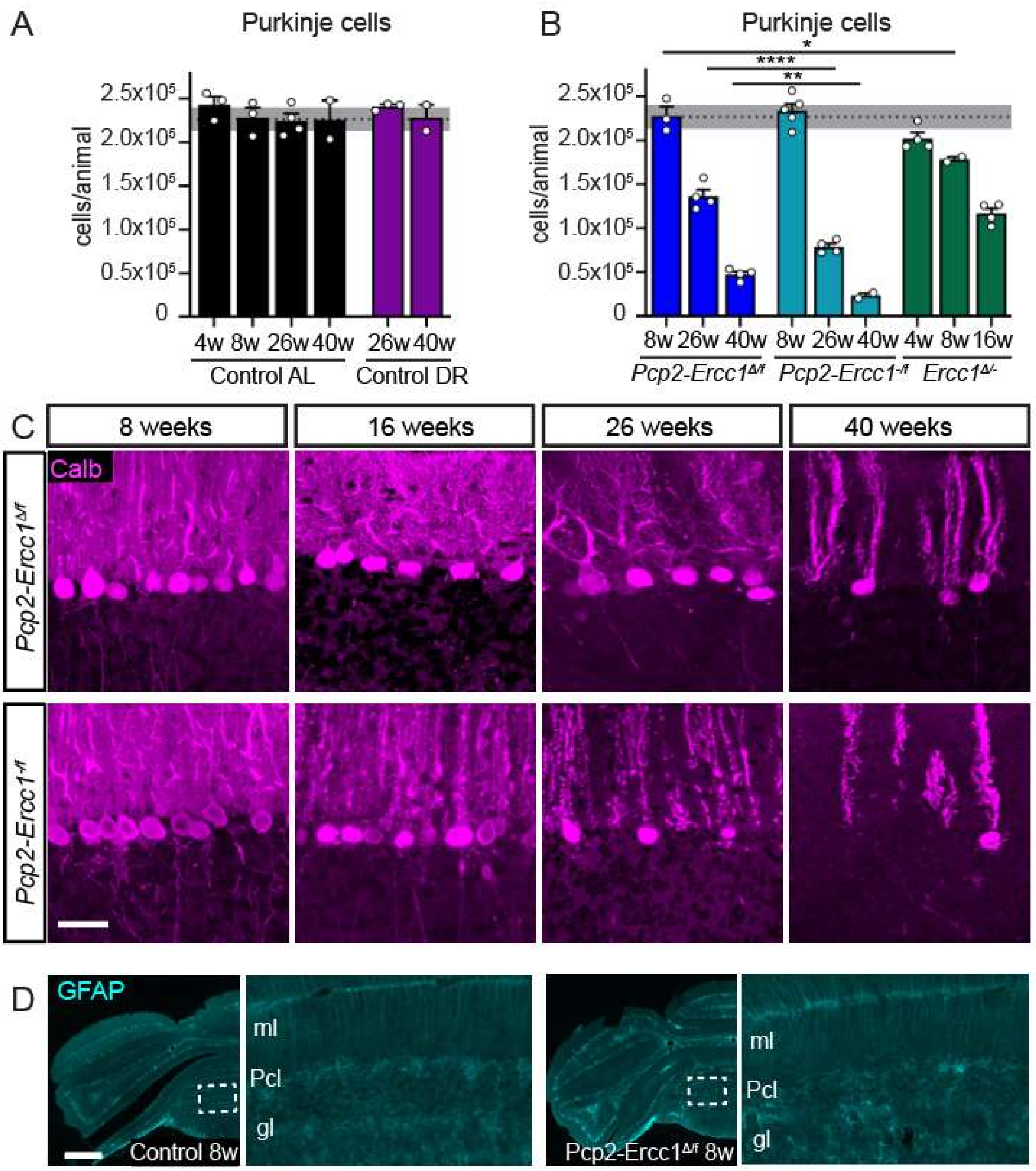

Supplementary Fig. S3. Faster Purkinje cell loss in Pcp2-*Ercc1*$^{-/f}$ mice compared to *Pcp2-Ercc1*$^{\Delta/f}$ mice

A. Bar graph of stereological Purkinje cell counts (means ± SE) in control animals of this study showing no effect of DR on the number of Purkinje cells (One-way ANOVA, $F(5, 11)=0.6030$; $P=0.6995$). The dotted line with shaded area indicates the mean and 95% confidence interval of pooled Purkinje cell values from 8-40 weeks-old AL control animals (n≥2/group).

B. Purkinje cell counts in AL-fed mice from three different ERCC1-deficient models at different ages, shown together with the mean and 95% confidence interval of control values (same as shown in a). *Pcp2-Ercc*$^{-/f}$ mice like *Pcp2-Ercc1*$^{\Delta/f}$ mice show unaltered numbers of Purkinje cells at 8 weeks of age, and show more severe Purkinje cell loss at 26 and 40 weeks (** P<0.01, **** P<0.0001, unpaired *t*-tests), indicative of faster Purkinje cell loss. Also note, that *Ercc1*$^{\Delta/-}$ mice at 8 weeks show reduced numbers of Purkinje cells compared to 8 weeks old *Pcp2-Ercc1*$^{\Delta/f}$ mice (*P<0.05, unpaired *t*-tests) and controls.

C. Exemplary images of calbindin immunostaining illustrating progressive Purkinje cell loss in *Pcp2-Ercc1*$^{\Delta/f}$ and *Pcp2-Ercc*$^{-/f}$ mice between 8 and 40 weeks of age.
D. Confocal images of GFAP immunofluorescence illustrating unaltered GFAP immunostaining in *Pcp2-Ercc1*$^{\Delta/f}$ cerebellum compared to control at 8 weeks of age (n≥2/group).
Scale bars: C, 25 µm; D, 1 mm
